# Establishment of a Machine Learning Model for the Risk Assessment of Perineural Invasion in Head and Neck Squamous Cell Carcinoma

**DOI:** 10.3390/ijms24108938

**Published:** 2023-05-18

**Authors:** Christopher Weusthof, Sebastian Burkart, Karl Semmelmayer, Fabian Stögbauer, Bohai Feng, Karam Khorani, Sebastian Bode, Peter Plinkert, Karim Plath, Jochen Hess

**Affiliations:** 1 Department of Otorhinolaryngology, Head and Neck Surgery, Section Experimental and Translational Head and Neck Oncology, Heidelberg University Hospital, 69120 Heidelberg, Germany; 2 Department of Otorhinolaryngology, Head and Neck Surgery, Klinikum rechts der Isar, Technical University Munich, 81675 Munich, Germany; 3 Department of Oral and Cranio-Maxillofacial Surgery, Heidelberg University Hospital, 69120 Heidelberg, Germany; 4 Institute of Pathology, School of Medicine, Technical University of Munich (TUM), 81675 Munich, Germany; 5 Research Group Molecular Mechanisms of Head and Neck Tumors, German Cancer Research Center (DKFZ), 69120 Heidelberg, Germany

**Keywords:** The Cancer Genome Atlas (TCGA), random forest, occult PNI, hypermethylation, somatic mutation, multiomics, single-cell sequencing

## Abstract

Perineural invasion is a prevalent pathological finding in head and neck squamous cell carcinoma and a risk factor for unfavorable survival. An adequate diagnosis of perineural invasion by pathologic examination is limited due to the availability of tumor samples from surgical resection, which can arise in cases of definitive nonsurgical treatment. To address this medical need, we established a random forest prediction model for the risk assessment of perineural invasion, including occult perineural invasion, and characterized distinct cellular and molecular features based on our new and extended classification. RNA sequencing data of head and neck squamous cell carcinoma from The Cancer Genome Atlas were used as a training cohort to identify differentially expressed genes that are associated with perineural invasion. A random forest classification model was established based on these differentially expressed genes and was validated by inspection of H&E-stained whole image slides. Differences in epigenetic regulation and the mutational landscape were detected by an integrative analysis of multiomics data and single-cell RNA-sequencing data were analyzed. We identified a 44-gene expression signature related to perineural invasion and enriched for genes mainly expressed in cancer cells according to single-cell RNA-sequencing data. A machine learning model was trained based on the expression pattern of the 44-gene set with the unique feature to predict occult perineural invasion. This extended classification model enabled a more accurate analysis of alterations in the mutational landscape and epigenetic regulation by DNA methylation as well as quantitative and qualitative differences in the cellular composition in the tumor microenvironment between head and neck squamous cell carcinoma with or without perineural invasion. In conclusion, the newly established model could not only complement histopathologic examination as an additional diagnostic tool but also guide the identification of new drug targets for therapeutic intervention in future clinical trials with head and neck squamous cell carcinoma patients at a higher risk for treatment failure due to perineural invasion.

## 1. Introduction

Haematogenic and lymphatic spread as well as local and perineural invasion (PNI) encompass the main routes for solid tumor dissemination. PNI is a common threat in multiple cancers, including head and neck squamous cell carcinoma (HNSCC), and serves as a prognostic indicator of unfavorable survival due to increased local recurrence rates and a shorter time to tumor relapse [1,2,3,4,5]. Therefore, HNSCC with PNI require a more aggressive and multimodal treatment for advanced stages including surgery, radiotherapy, and chemotherapy [6]. Based on a histopathological inspection, PNI is broadly defined as the extension of cancer cells around, into, or through nerves. Historically, it has been thought to be a unilateral process dependent upon cancer invasion with a tumor invading the perineural space through the path of least resistance. However, more recent studies have elucidated a more complex picture of mutual cancer–neuron interaction in facilitating PNI with a crucial role of the perineural microenvironment involving distinct molecular and cellular constituents of peripheral nerves [7].

Despite the emerging clinical relevance, the determination of PNI by the histopathological examination of entire histological slides remains a major challenge and is subject to a wide variance of reported incidence rates for HNSCC (between 6% and 90%). The high variance can be explained in part because PNI can involve both small and large nerves with sporadic distribution patterns in sometimes extremely large tissue samples, making accurate pathological assessment a time consuming and tedious task. To solve this problem, several recent studies focused on computational approaches to extract nerves and PNI from histologically stained whole-slide images, utilizing deep learning networks or artificial-intelligence-based classifiers (Table 1). Though a computer-assisted diagnosis appears feasible, limitations to these studies are the small sample sizes and the lack of independent validation in larger clinical cohorts, so far. In addition, these approaches critically depend on whole-slide images from a surgical resection of the primary tumor, which are not available for cases treated with definitive radio-/chemotherapy (RCT), where pathological examination is hampered by small tumor specimens from biopsies [8,9]. Especially in early and in locoregionally advanced stages of laryngeal squamous cell carcinoma, definitive RCT represents the favored treatment in many clinical centers [10,11,12]. In this context, it is also worth noting that currently, no therapeutic intervention exists for PNI in HNSCC patients. These issues illustrate the urgent medical need for reliable molecular classifiers to support diagnostic PNI assessment and prognostic risk prediction, and to explore the exact underlying molecular principles.

In the past, most studies focused on individual genes or signaling pathways, and only a few explored PNI-related molecular differences at the omics level (Table 1). The study by Saidak et al. identified a PNI-related gene expression profile enriched in muscle genes and demonstrated increased activation levels of AKT/PKB and mTOR kinases for HNSCC with PNI [13]. Zhang et al. established a PNI-associated coexpression module consisting of genes (*n* = 357) that functioned in processes such as extracellular matrix remodeling, collagen catabolic processes, and cell adhesion [14]. The analysis of single-cell RNA sequencing (scRNA-seq) data revealed that the expression of genes in the PNI-associated module resembled functional states of the epithelial-to-mesenchymal transition (EMT), metastasis, and invasion in cancer cells. Both studies were limited to patients with a confirmed PNI status and excluded cases lacking a histopathological assessment [13,14]. Hence, the potential of these gene expression profile or module to identify occult PNI for HNSCC patients without a surgical resection of the primary tumor or a PNI-negative assessment according to established diagnostic criteria is unknown. The latter issue might be of particular clinical relevance considering the data of a more recent study on the spatial and transcriptomic analysis of PNI in oral cancer [15]. In that study, patients with nerves closer to the tumor had poor outcomes even if diagnosed as PNI-negative using established histopathological criteria.

**Table 1:** Literature review presenting in total 6 related studies and pointing out different study designs and results.

Samples	Dataset (Cases)	Deliverables	Reference
OSCC	H&E-stained whole-slide images (training set *n* = 20, validation set *n* = 60)	Simultaneous segmentation of microvessels and nerves	Neural Comput &Applic 32, 9915–9928 (2020) [16]
HNSCC	H&E-stained whole-slide images (*n* = 334)	Segmentation of nerves and PNI	PMID: 36496513 [17]
OSCC	H&E-stained whole-slide images (training set = 80, validation set = 10)	PNI classifier	PMID: 36353548 [18]
HNSCC	RNA-seq and clinical data (TCGA. *n* = 351), scRNA-seq (GSE103322, *n* = 18)	PNI-associated coexpression module with 12 hub genes	PMID: 31214495 [14]
HNSCC	Multiomics and clinical data (TCGA, *n* = 361)	PNI-related gene expression profile (263 genes)	PMID: 30409320 [13]
OSCC	H&E- an ICH- stained whole-slide images (*n* = 142), NanoString Spatial Profiling	Spatial and transcriptomic analysis	PMID: 35819260 [15]

The main objective of this study was the training of a machine learning (ML) model based on a PNI-related gene set to enable the accurate prediction of occult PNI, which could complement pathological examination, and to provide new insights into molecular alterations which are associated with the presence of PNI in HNSCC.

## 2. Results

### 2.1. PNI as an Independent Prognostic Factor

To confirm the clinical relevance of perineural invasion (PNI) as an independent prognostic factor for poor outcome, we performed a survival analysis based on the histopathologic PNI status, including all PNI− and PNI+ tumors of the TCGA-HNSC cohort (*n* = 317) with accessible survival data. Kaplan–Meier graphs and log-rank tests demonstrated significant differences in 5-year overall survival (OS), disease-specific survival (DSS), and progression-free intervals (PFI) for this cohort (Supplemental Figure S1A). A subgroup analysis confirmed significant differences in 5-year survival for oral squamous cell carcinoma and laryngeal squamous cell carcinoma but not for oropharyngeal squamous cell carcinoma (Supplemental Figure S1B). A crosstab analysis revealed a significant association of PNI+ tumors with oral squamous cell carcinoma (*p* = 1.14 × 10^−5^), tumor size (*p* = 8.93 × 10^−5^), lymph node metastasis (*p* = 3.5 × 10^−6^), extracapsular spread (*p* = 3.64 × 10^−6^), and angiolymphatic invasion (*p* = 9.94 × 10^−6^) (Supplemental Table S2).

### 2.2. PNI-Related Gene Expression Signature

**Figure 1:**
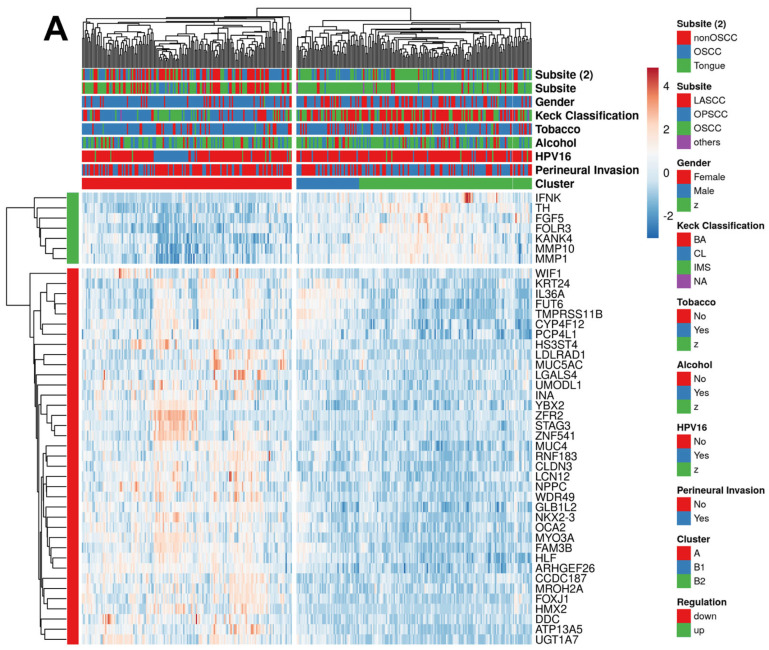

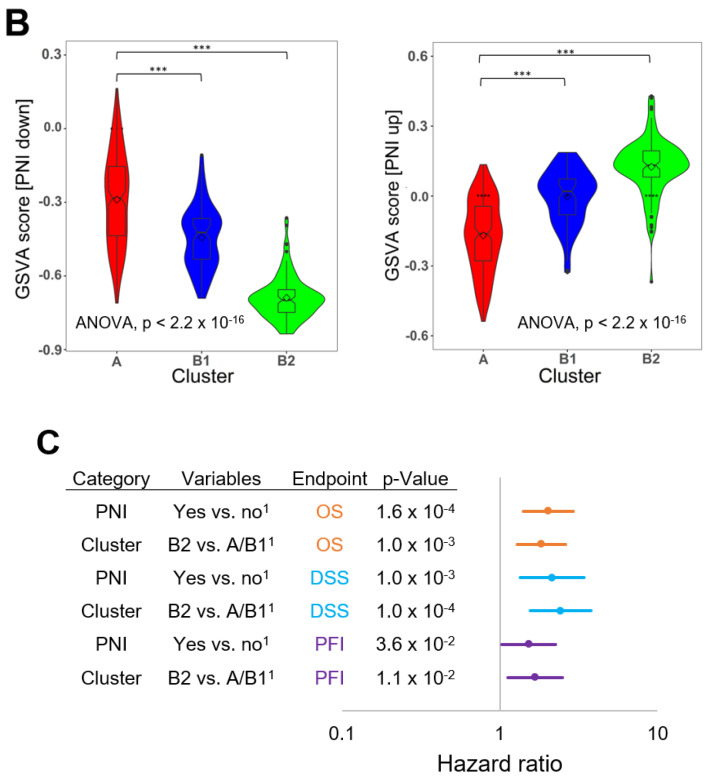
Identification of subgroups in TCGA-HNSC with annotated PNI status and distinct clinical and prognostic features based on the PNI-related 44-gene set. (**A**) A heatmap shows an unsupervised hierarchical clustering of tumors from TCGA-HNSC with an annotated PNI status (*n = 348*) based on gene expression data of the PNI-related 44-gene signature and demonstrates two main groups: cluster A (red, enriched for PNI−) and cluster B, subdivided in subcluster B1 (blue) and B2 (green, enriched for PNI+). (**B**) Violin plots illustrate a significant difference in GSVA scores of up or downregulated DEGs of the PNI-related 44-gene signature between cluster A (red) and subclusters B1 (blue) and B2 (green). (**C**) Forest plot for the 5-year OS, DSS, and PFI of patients from TCGA-HNSC with an annotated PNI status based on a univariate Cox regression model stratified by either the pathological PNI status or classified by the PNI-related 44-gene signature. ^1^ Reference group. * *p* < 0.05, *** *p* < 0.0005, **** *p* < 0.00005, as determined by an ANOVA and Tukey’s post hoc test.

Next, we addressed the question of whether differences at the transcript level enabled a risk assessment of PNI (Supplemental Figure S2). An analysis of bulk RNA-seq data from TCGA-HNSC was performed using the limma and edgeR packages in R and revealed 60 common differentially expressed genes (DEGs) between tumors with or without annotated PNI (Supplemental Figure S3A–C; Supplemental Table S3). An unsupervised hierarchical clustering based on the transcript levels of these DEGs revealed two main clusters, which were enriched for either PNI− (cluster A) or PNI+ (cluster B) (Supplemental Figure S3D). However, the cluster analysis highlighted a set of upregulated DEGs (*n = 16*) in PNI+ tumors, which were related to muscle tissue and strongly enriched for tongue squamous cell carcinoma (Supplemental Figure S3D), indicating a bias by the anatomical subsite. The removal of these 16-gene set revealed a final PNI-related 44-gene signature of which 7 genes were upregulated and 37 genes were down-regulated in PNI+ as compared to PNI− HNSCC. The unsupervised hierarchical clustering based on transcript levels of the PNI-related 44-gene set confirmed two main clusters A and B, the latter subdivided in two subclusters B1 and B2 (Figure 1A). PNI+ tumors were strongly enriched in subcluster B2 (Supplemental Table S4), and differences in gene set variation analysis scores for up- (ANOVA *p* < 2.2 × 10^−16^) or downregulated (ANOVA *p* < 2.2 × 10^−16^) genes of the PNI-related 44-gene set were highly significant between subcluster B2 and cluster A or subcluster B1 (Figure 1B). HPV16-positive oropharyngeal squamous cell carcinoma (HPV16+ OPSCC) was significantly enriched in cluster A, while no major difference was evident for other risk factors (tobacco and alcohol, Supplemental Table S4). Concerning the classification by Keck et al. [19], tumors with a basal subtype (BA) were more often in subcluster B2 while tumors with a classical subtype (CL) were more frequent in cluster A (*p* < 2.2 × 10^−16^) (Supplemental Table S4). A survival analysis confirmed a significant difference in overall (OS, HR = 1.838, *p* = 1.0 × 10^−3^), disease-specific survival (DSS, HR = 2.429, *p* = 1.0× 10^−4^), and progression-free interval (PFI, HR = 1.671, *p* = 1.1 × 10^−2^) between cases of clusters A/B1 and B2 similar to the stratification by the pathological PNI status (Figure 1C). In summary, the PNI-related 44-gene signature enabled the stratification of the TCGA-HNSC cohort with an annotated PNI status in molecularly defined groups with distinct clinical features and prognosis.

### 2.3. Validation in TCGA-HNSC without Annotated PNI Status and Independent HNSCC Cohorts

**Figure 2:**
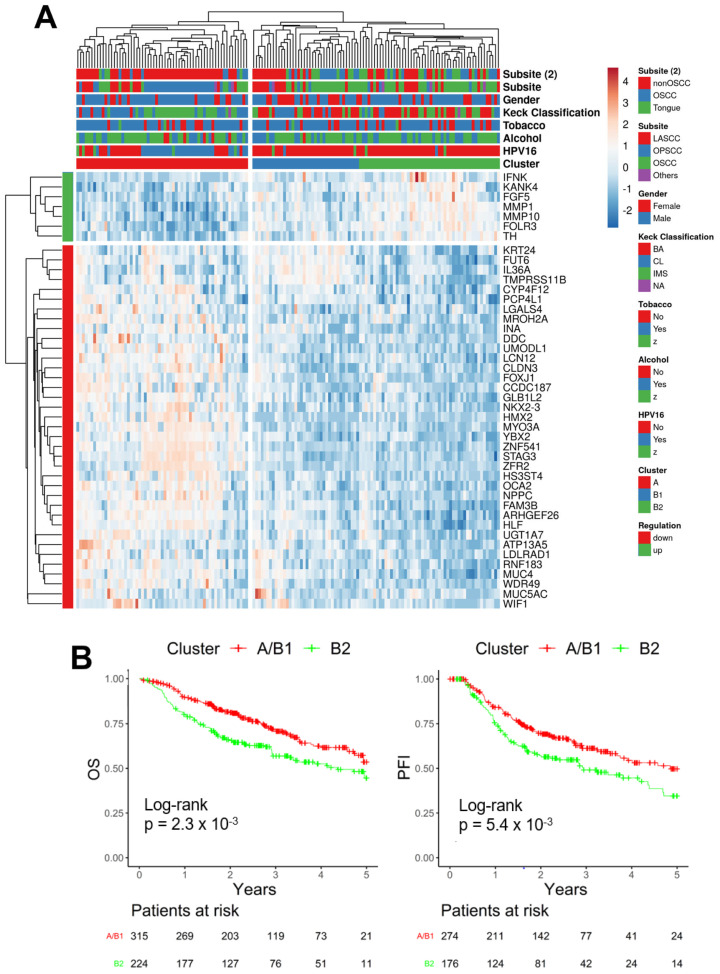
Expression of the PNI-related 44-gene set identifies clinically relevant subgroups in HNSCC cohorts without annotated PNI status. (**A**) A heatmap shows an unsupervised hierarchical clustering based on gene expression data of the PNI-related 44-gene signature and confirms two main clusters A (red) and B, the latter subdivided into sub-cluster B1 (blue) and B2 (green) for tumors from TCGA-HNSC without an annotated PNI status (*n* = 152). (**B**) Kaplan–Meier plots for 5-year overall survival (OS, left) and 5-year progression-free intervals (PFI, right) for patients of TCGA-HNSC without an annotated PNI status and independent HNSCC cohorts (GSE65858, GSE41613, GSE117973), which were classified into cluster A/subcluster B1 (red) or subcluster B2 (green). Number of patients at risk at the indicated time points are given below.

To confirm the association of the PNI-related 44-gene signature with clinical features and its prognostic value, its expression pattern was investigated for the TCGA-HNSC cohort without annotated PNI status (TCGA-HNSC NA, *n* = 149) and for independent HNSCC cohorts (GSE117973, GSE65858, GSE41613). An unsupervised hierarchical clustering revealed a similar stratification into cluster A and subclusters B1 and B2 in all validation cohorts (Figure 2A, Supplemental Figure S4A–C).

As for the training cohort, a significant enrichment was evident for HPV16+ tumors in cluster A for all cohorts for which the HPV16 status was available (Supplemental Table S5). In addition, Kaplan–Meier plots and log-rank tests for the combined validation cohorts of HNSCC revealed a significant difference between clusters A/B1 and B2 for OS (*p* = 2.3 × 10^−3^) and PFI (*p* = 5.4 × 10^−3^) (Figure 2B). These data confirm the prognostic value of the PNI-related 44-gene signature in HNSCC for which a histopathological PNI status is not available.

### 2.4. Single-Cell RNA-Sequencing Analysis of the PNI-Related 44-Gene Signature

**Figure 3:**
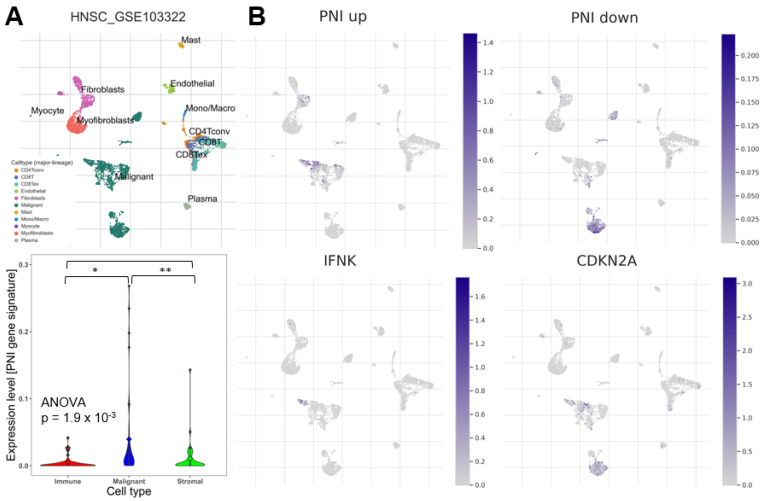

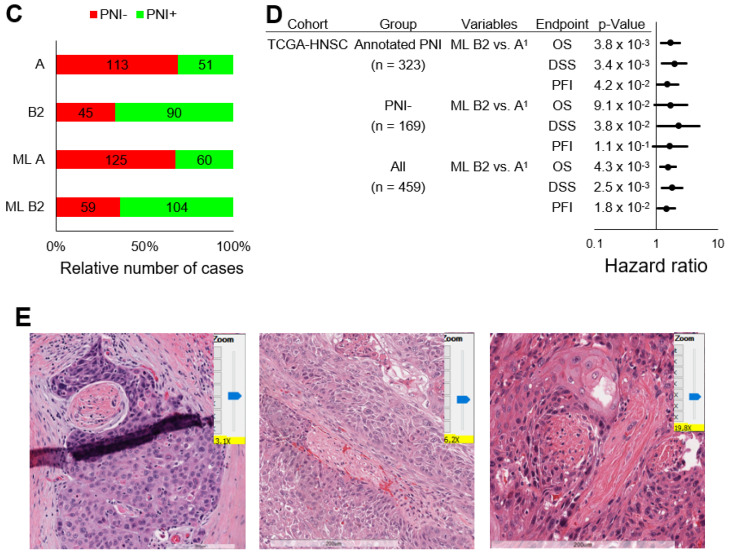
Prominent expression of the PNI-related 44-gene signature in cancer cells and establishment of a PNI classifier by a random forest model. (**A**) UMAP plot visualizing the different cell types given in GSE103322 [17], with included figure legend (malignant, endothelial, mast cells, plasma, myocyte, myofibroblasts, fibroblasts, CD4, CD8, monocytes, macrophages). Violin plot demonstrating the statistical significance of malignant cell scores (blue) compared to immune (red) and stromal (green) cell scores based on the 44-gene set. * *p* < 0.05, ** *p* < 0.005 as determined by an ANOVA and Tukey’s post hoc test. (**B**) UMAP plots for the 37 downregulated (PNI down) and 7 upregulated (PNI up) DEGs of the 44-gene signature as well as CDKN2A and IFNK illustrating the expression of these genes in distinct cell types in GSE103322 [17]. (**C**) The bar plot summarizes the relative frequency of PNI− (red) or PNI+ (green) tumors from TCGA-HNSC with an annotated PNI status, which were classified as either cluster A and subcluster B2 by the PNI-related 44-gene signature or ML A or ML B by the ML model. (**D**) Forest plot for 5-year OS, DSS, and PFI based on univariate Cox regression models for patients from the indicated groups of TCGA-HNSC, which were stratified by the ML model into ML A or B. ^1^ Reference group. (**E**) Representative pictures of digital images for H&E-stained slides from the Cancer Digital Slide Archive of TCGA-HNSC confirms the presence of PNI for clinically PNI-annotated tumors and tumors without PNI annotation, which were predicted as ML B2 by the random forest model.

Single-cell RNA-sequencing fundamentally maps the cellular heterogeneity of tumors and their microenvironment. In a recent study, Zhang et al. reported a prominent expression of key genes from a PNI-associated coexpression module in fibroblasts and concluded that these nonmalignant cells of the tumor microenvironment played an important role in PNI [14]. To address the question of whether the genes of our newly defined PNI-related 44-gene signature were also expressed in both malignant and nonmalignant cells, including fibroblasts, we performed a single-cell RNA-sequencing analysis utilizing data from GSE103322 and the TISCH2 online tool [20]. This analysis demonstrated that the 37 downregulated genes (including *CDKN2A*) as well as the 7 upregulated genes (including *IFNK*) of the PNI-related 44-gene signature were preferably expressed in different subpopulations of malignant cells. In contrast, expression values were significantly lower in stromal cells, including fibroblasts, and were barely detectable in immune cells (Figure 3A,B). In summary, these data indicate a cancer-cell intrinsic gene regulatory program in the establishment and maintenance of PNI, which might be modulated by cellular signals and the matrix composition of the tumor microenvironment.

### 2.5. Establishment of a PNI-Related Machine Learning Model for Occult PNI

The next task was to establish a machine learning (ML) model to further improve prognostic and diagnostic risk prediction by a molecular classification based on the expression of the PNI-related 44-gene signature. The TCGA-HNSC cohort with annotated PNI status was split into a training (80%) and test (20%) dataset and clusters A (enriched for PNI−) versus subcluster B2 (enriched for PNI+) was selected as category for the training of individual machine learning models. We excluded subcluster B1 for the training of the classification model due to the almost balanced distribution of PNI− and PNI+ cases (Supplemental Table S4). Three commonly used classification models (random forest, neural network, and logistic regression [21,22]) revealed a comparable prediction accuracy for the PNI status with similar positive and negative prediction values, but slight differences in sensitivity and specificity (Supplemental Table S6). Finally, we selected the random forest model, which performed slightly better as compared to the other two models, for further analysis (Supplemental Figure S4D–F, Supplemental Table S6). It is worth noting that the moderate values for the prediction accuracy (0.68) and AUC (0.604) might be explained by the expected frequency of false-negative PNI classifications for TCGA-HNSC. Based on the random forest model, tumors of TCGA-HNSC were classified into ML A (enriched for PNI−) and ML B2 (enriched for PNI+) (Figure 3C). Again, ML B2 classified HNSCC exhibited an unfavorable overall survival (OS, HR = 1.540, *p* = 4.3 × 10^−3^), disease-specific survival (DSS, HR = 1.803, *p* = 2.5 × 10^−3^), and progression-free interval (PFI, HR = 1.473, *p* = 1.8 × 10^−2^) compared to its ML A counterparts (Figure 3D), and ML B2 served as an independent risk factor for unfavorable survival in a multivariate Cox regression analysis (Table S7). Strikingly, a substantial number of PNI− tumors based on a histopathological examination (*n = 60*) was assigned by the model as ML B2. These ML B2/PNI− tumors had a significantly worse disease-specific survival (DSS, HR = 2.288, *p* = 3.8 × 10^−2^) and showed a clear trend towards unfavorable overall survival (OS, HR = 1.711, *p* = 9.1 × 10^−2^) and progression-free interval (PFI, HR = 1.669, *p* = 1.1 × 10^−1^) compared to ML A/PNI− tumors (Figure 3D). These data indicated that our newly established classifier enabled the identification of occult PNI in HNSCC, which were classified as PNI− after the histopathological examination. To further support this assumption, we selected twelve ML B2 classified tumors from TCGA-HNSC with a PNI− status (according to clinical annotation, *n = 6*) or missing information on the PNI status (PNI NA, *n = 6*) for which H&E-stained whole-slide images were available from the Cancer Digital Slide Archive (Supplemental Table S8). Despite the limited number of whole-slide images available for individual tumors (range 2–4), six tumors showed histological evidence for PNI after a re-evaluation (Figure 3E).

The random forest model was also applied on bulk RNA-seq data from other solid tumors of TCGA (Supplemental Table S9). As for HNSCC, ML B2 served as an unfavorable risk factor for overall survival, which reached statistical significance for cervical squamous cell carcinoma (CESC) and exhibited a clear trend for adenocarcinomas of the lung (LUAD), pancreas (PAAD), and colon (COAD, Supplemental Figure S5A). In line with this finding, an unsupervised hierarchical clustering of TCGA-CESC based on the expression values of the PNI-related 44-gene set demonstrated a similar stratification of tumors into cluster A and subclusters B1 and B2 (Supplemental Figure S5B).

### 2.6. PNI-Related Alterations in the Mutational Landscape

**Figure 4:**
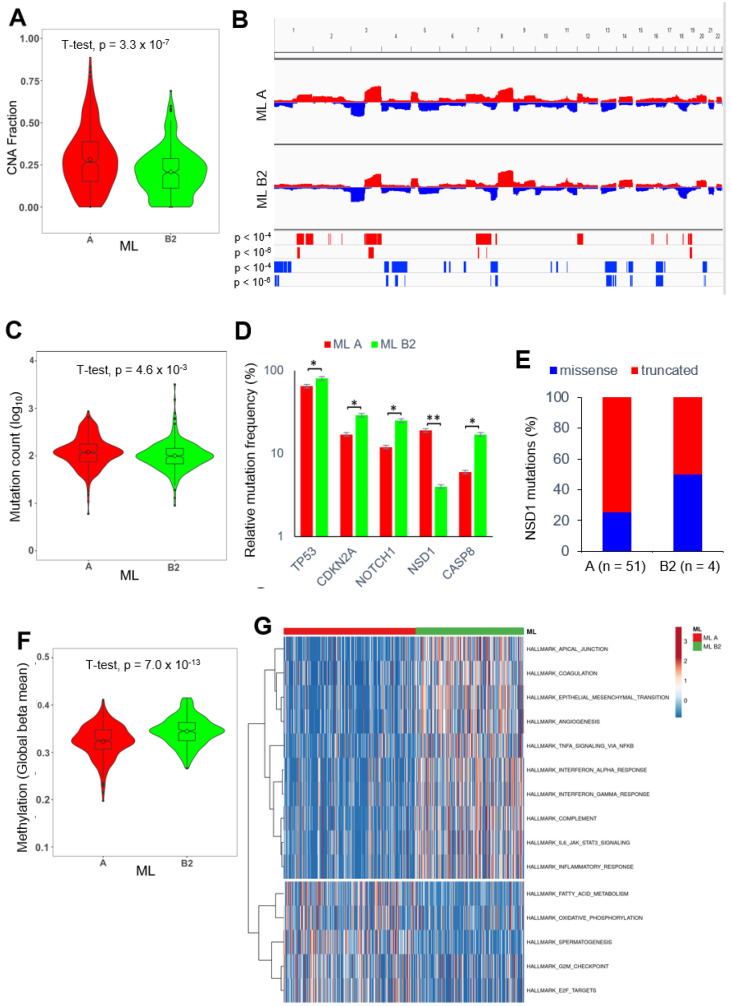
Differences in the mutational landscape, global DNA methylation, and gene regulatory networks between ML A and ML B tumors. (**A**) Violin plot demonstrates a significant difference in the fraction of altered genome between ML A (red) and ML B2 (green) tumors. (**B**) CNA plots illustrate copy number gains (red) and losses (blue) for ML A and ML B2 tumors and demonstrate hot-spot regions with significant differences as calculated by Fisher’s exact test. (**C**) Violin plot demonstrates a significant difference in somatic mutation counts between ML A (red) and ML B2 (green) tumors. (**D**) Bar plot shows MutSig genes with a significant difference in somatic mutation frequency between ML A (red) and ML B2 (green) tumors. (**E**) Bar plot shows the relative frequency of tumors with either missense (blue) or truncating (red) *NSD1* mutations for ML A and ML B2 tumors. (**F**) Violin plot demonstrates a significant difference in global DNA methylation between ML A (red) and ML B2 (green) tumors. (**G**) Heatmap illustrates an unsupervised hierarchical clustering based on GSVA scores for indicated HALLMARK gene sets from MSigDB. * *p* < 0.05, ** *p* < 0.005 as determined by a chi-square test.

Next, we addressed the question of whether the PNI classification of HNSCC by the random forest model was related to differences in the mutational landscape. The analysis of global copy number alterations (CNAs) revealed a highly significant increase in the global copy number alteration fraction for ML A compared to ML B2 for TCGA-HNSC (*p* = 3.3 × 10^−7^) and identified distinct hotspot regions of copy number gain or loss (Figure 4A, B). ML A head and neck squamous cell carcinoma were significantly enriched for copy number losses of chromosomes 1p, 4, 13, 14q, 16q, and 20q, and gains in chromosomes 1q, 3q, 7q, and 19p, while ML B2 tumors exhibited a more prominent loss at chromosome 8p (Figure 4B, Supplemental Figures S6A,B and S7A,B). Interestingly, some genes of the PNI-related 44-gene set were encoded at the affected genomic loci, which might explain their differential expression between ML A and ML B2 tumors (Supplemental Table S3). Moreover, total somatic mutation counts were significantly higher for ML A HNSCC compared to their ML B2 counterparts (*p* = 4.6 × 10^−3^) (Figure 4C), and several MutSig genes demonstrated a significant difference in somatic mutation frequency between both groups (Figure 4D). The most prominent difference was detected for *NSD1*, which exhibited a significant enrichment of somatic mutations for ML A.

Recent studies reported truncating *NSD1* mutations in laryngeal squamous cell carcinoma, which were accompanied with a better prognosis and global DNA hypomethylation [23,24]. As the majority of somatic *NSD1* mutations in ML A tumors were truncating mutations (Figure 4E), we analyzed the global DNA methylation pattern for TCGA-HNSC. Indeed, a highly significant decrease in global beta mean values was evident for ML A compared to ML B2 tumors (Figure 4F).

### 2.7. PNI-Related Alterations in the Immune Landscape

To address the potential impact of distinct immune cell subsets in the tumor microenvironment on the PNI risk prediction, the inferred abundance for immune cell and other stromal cells from xCell, CIBERSORTx, and Kassandra was compared between ML A and ML B2 tumors. This analysis demonstrated significantly higher number of B cells and T cells, in particular CD4 T cells, in ML A compared to ML B2, while endothelial cells and fibroblasts were significantly more abundant in ML B2 compared to ML A (Supplemental Figure S8).

### 2.8. PNI-Related Alterations in Gene Regulatory Networks and Pathway Activities

Finally, we explored differences in pathway activities between ML A and ML B2 classified tumors of TCGA-HNSC based on gene set variation analysis (GSVA) scores for the hallmark gene sets from MSigDB (Supplemental Table S10). Top ranked gene sets indicated an upregulation of processes related to the epithelial-to-mesenchymal transition (EMT) and inflammation in ML B2 tumors, while ML A tumors showed the upregulation of genes related to metabolic processes, E2F targets, and cell cycle regulation (Figure 4G).

## 3. Discussion

PNI represents a complex and mutual crosstalk between cancer cells and components of peripheral nerves, which is partially triggered by secreted neurotrophic and other growth factors affecting transcription, translation, and cytoskeletal reorganization in cancer and neuronal cells [25,26,27,28,29,30,31,32], illustrating the importance of nerves in cancer cell dissemination [33,34,35]. Despite the clinical relevance of PNI as a prognostic risk factor for most solid cancers, including HNSCC, its histopathological assessment is hampered by the limited availability of tumor tissue for pathological examination in cases of definitive treatment with radio- and chemotherapy [10,11,12]. Hence, a reliable risk model for PNI based on molecular characteristics is an unmet medical need to improve the diagnosis and treatment decision making for primary HNSCC but also other solid tumors. It is also instrumental for a better understanding of the basic principles in the mutual interaction between cancer cells and neurons to establish new strategies for therapeutic intervention aiming to prevent cancer cell dissemination as a main cause of treatment failure and cancer-related mortality [36,37].

In this study, we established a PNI-related 44-gene signature which enabled the classification of TCGA-HNSC as a training cohort and several independent HNSCC validation cohorts into different subgroups with distinct clinical and molecular features as well as prognosis. Based on the transcript levels of the PNI-related 44-gene signature, we trained a random forest model which offered several novel and attractive options: (i) the identification of primary HNSCC with occult PNI despite a false-negative histopathological assessment or the limited availability of biomaterial, (ii) an unprecedented in-depth molecular analysis of cancer-cell intrinsic and extrinsic alterations, such as the cellular composition of the tumor microenvironment as a driving force of PNI, and (iii) the PNI-related prognostic risk prediction in other solid cancers beyond HNSCC.

In 2018, Saidak et al. utilized transcriptome data from TCGA-HNSC to establish a PNI-related gene signature and to explore the molecular mechanisms involved in PNI [13]. This gene signature was highly enriched for genes related to muscle differentiation and function and most likely resembled the high prevalence of tongue squamous cell carcinomas in the PNI+ group of HNSCC. Though an altered expression of muscle-related genes in HNSCC cancer cells and a potential impact on PNI could not be formally excluded [37], we assumed that their association with the PNI status was biased by the unequal distribution of tumor subsites and therefore we excluded sixteen muscle-related genes from further analysis. This assumption was supported by PNI-related gene sets from other solid tumors, e.g., gastric cancer or pancreatic ductal adenocarcinoma (PDAC), which were not enriched for muscle-related genes [38,39]. In addition, Lee et al. proposed that only *ACTA1* was related to PNI for tongue squamous cell carcinoma considering an actin-associated gene set [40].

In another study, Zhang et al. identified PNI-associated gene coexpression modules [14]. One module containing 357 genes with 12 hub genes was correlated with gene signatures related to the epithelial-to-mesenchymal transition, metastases, and invasion. Among nonmalignant cells, fibroblasts had a relatively high expression of hub genes, indicating a potential role of cancer-associated fibroblasts for PNI. Interestingly, 26 genes of the PNI-associated gene coexpression module of that study were also part of our 44-gene signature and we also identified overlapping pathway activities (e.g., epithelial-to-mesenchymal transition, angiogenesis) for ML B tumors classified by the random forest model. However, single cell an RNA-sequencing analysis demonstrated a more prominent expression of the 44-gene signature in malignant cells and only minor or barely detectable expression in stromal cells, including fibroblasts. Though these data do not formally exclude a critical role of fibroblasts, they indicate a cancer cell intrinsic gene regulatory program in the formation and maintenance of PNI, which might be modulated by a direct or indirect interaction with matrix or cellular components of the tumor microenvironment.

In a more recent study, a closer nerve–tumor distance and larger nerves in the tumor bulk were identified as predictors for unfavorable survival of oral cancer even if diagnosed as PNI-negative tumors [15]. Spatial transcriptomic analyses illuminated specific patterns of nerve–cancer interaction suggesting a cancer-induced modulation of neurogenesis. This assumption was further supported by Amit et al. who demonstrated that the loss of p53 in cancer cells led to neuronal reprogramming and axonogenesis in a mouse model and that the somatic *TP53* mutation status was associated with nerve density in a retrospective study with oral cancers [25]. Hence, it is worth speculating that the strong inverse association between PNI and HPV in this and previous studies [14] is due to the lack of somatic TP53 mutations in most HPV16+ oropharyngeal squamous cell carcinoma. A prominent p16 protein expression (encoded by *CDKN2A*), which is a surrogate biomarker for HPV16+ tumors. might represent another reason for low PNI frequency in HPV16+ oropharyngeal squamous cell carcinoma. *CDKN2A* is one of the MutSig genes with a significantly higher relative frequency of somatic mutations in ML B2 and limited p16^INK4A^ function due to *CDKN2A* mutation or deletion was associated with perineural invasion in nonsmoking and nondrinking oral squamous cell carcinoma patients [38]. Moreover, the DNA methylation of the *CDKN2A* promoter was a more frequent event in aggressive prostatic tumors with PNI [41]. Several other studies demonstrated an association between the epigenetic regulation of specific candidate genes by altered DNA methylation and the development of PNI [42,43,44,45,46,47,48,49,50]. Still, the DNA methylation status in head and neck squamous cell carcinoma has not yet been studied sufficiently [50]. Consistent with these studies, genome-wide DNA hypomethylation was evident in ML A HNSCC at least in part due to truncating *NSD1* mutations. In summary, epigenetic regulation by DNA methylation (e.g., *CDKN2A*) could represent a key driver affecting the nerve–cancer crosstalk leading to PNI in HNSCC and serve as a promising drug target for therapeutic intervention in the cancer dissemination via nerve tracks [51].

Matrix metalloproteinases (MMPs) are well understood to play a part in tumor invasion and metastases being able to cause a degradation of the extracellular matrix (ECM) [48]. *MMP1*, which is part of our 44-gene set and is produced in malignant cells, initiates a signaling pathway via neuronal protease-activated receptor 1 (*PAR1*) and carcinoma neurokinin 1 receptor (*NK1R*). This is known to play a crucial role in tumor progression and development of PNI [52]. In addition, *MMP2* and *MMP9* are well known to be involved in metastases and tumor cell dissemination of several tumors [38,53,54]. So far, any attempts to establish MMP inhibitors in tumor therapy proved unsuccessful in terms of survival and because of toxicity [49]. This failure might be explained by the use of broad-spectrum MMP inhibitors, which consequently also counteracted antitumorigenic MMPs [55]. However, studies on novel highly specific MMP inhibitors targeting noncatalytic domains have shown promising approaches and, as we can demonstrate with our results, may also be of great importance in the future regarding tumor dissemination via PNI in HNSCC [56,57].

One limitation of this study includes the retrospective study design. In addition, genome-wide transcriptome data were used to identify the PNI-related 44-gene set and to train the random forest model, which are not determined in routine clinical practice for HNSCC patients. However, our study provides compelling evidence that a quantitative assessment of gene expression for a smaller number of selected genes could be utilized to predict the risk for PNI, i.e., by quantitative RT-PCR or panel RNA-sequencing. For a more feasible clinical application, future prospective studies could equally attempt to achieve a risk assessment for PNI based on single genes of the 44-gene signature with a good classification of patients similar to our random forest model. Additionally, by performing a differential gene expression analysis, we used a rather conservative approach to set up the study. Using emerging methods (i.e., deep learning network), it might be possible to further improve our model by integrating multiple clinical, cellular, and molecular features in future studies. Another limitation is linked to the use of publicly available databases and their clinical annotations, which represents a risk for false-negative cases from a wrong annotation of the PNI status. A false-negative PNI status could have an impact on the identification of the PNI-related 44-gene signature. Nevertheless, we can assume that a misclassification of the clinical PNI status can be compensated in our random forest model. Moreover, the more precise prediction of the PNI status by the random forest model, including a more precise assessment of cases with occult PNI and regardless of tumor sample size, offers the unique possibility of analyzing molecular and cellular differences more accurately, which could provide new insights and correlations in the development of PNI in HNSCC but also in other solid tumors.

## 4. Materials and Methods

### 4.1. Data Collection and Key Resources

Detailed information about data collection of publicly available data and the use of online tools and software is summarized in Supplemental Table S1. Statistical analyses were performed in R software Version 3.5.3 [58] and SPSS Version 26 [59] with *p* < 0.05 as the significance level. For the head and neck squamous cell carcinoma cohort from The Cancer Genome Atlas (TCGA-HNSC, *n* = 500) which served as the training cohort in machine learning, RNA expression and clinical data were downloaded from https://portal.gdc.cancer.gov (accessed on 3 May 2019). As validation cohorts, we used three independent cohorts with transcriptome data from primary head and neck squamous cell carcinoma (GSE65858, GSE41613, and GSE117973) and other solid tumors from TCGA.

### 4.2. Survival Analysis

A Kaplan–Meier analysis was performed to estimate overall (OS), disease-specific survival (DSS), and progression-free interval (PFI). Log-rank tests were conducted to calculate statistically significant differences in survival. Univariate and multivariate Cox regression models were performed by SPSS and R. Patients with a secondary tumor, neoadjuvant treatment, an event in PFI or DSS within the first three months, a history of other malignancies, and an M1 status were excluded from the survival analysis.

### 4.3. Differential Gene Expression Analysis

The analysis of differentially expressed genes (DEGs) was performed using the packages “limma” and “edgeR” [60,61]. The voom transformation was executed for the DEG analysis with “limma”. DEGs between tumors with a positive or negative PNI status were considered significant with FDR < 0.05 and |log2-fold change| > 1. Common DEGs of the analysis with limma or edgeR were identified by a Venn diagram [62].

### 4.4. Unsupervised Hierarchical Clustering

Transcriptome count data of genes were ln(x + 1)-transformed and clustered using the correlation distance and average linkage. ClustVis, a web tool for visualizing multivariate data, was utilized for unsupervised hierarchical clustering and to visualize data in a heatmap [63].

### 4.5. Single-Cell RNA-Sequencing Analysis

For the analysis of single-cell RNA sequencing data, we used the online tool “Tumor Immune Single-cell Hub 2 (TISCH2)”, applied our gene signature, and selected single genes (*CDKN2A, IFNK*) from two different datasets (GSE103322 [17]) [64]. We downloaded the expression data for malignant, stromal, and immune cells for the statistical analysis in R on 14 January 2023.

### 4.6. Machine Learning Models and Review of Digital Slides

The “caret” package in R was used to train a machine learning (ML) model for clusters A and subcluster B2 based on the transcript values (FPKM) of the PNI-related 44-gene signature. To create a best performing model for the risk prediction of PNI, we used cluster A and subcluster B2 but excluded subcluster B1 with an almost balanced ratio of PNI− and PNI+ tumors. A cross-validation was used for parameter tuning with a tuning length of 150 (80% training set and 20% validation set). We computed a confusion matrix and plotted ROC curves for the three used classifiers (random forest, neural networks, logistic regression). Finally, the random forest model was selected for further analysis, which also had the advantage of dealing with relatively small datasets and binary variables compared to neural networks. The trained random forest model was applied to other solid tumors from TCGA, and some cohorts (BRCA, GBM, KIRC, LIHC, PRAD, SKCM, TGCT, THCA) were excluded from the further analysis because of an unbalanced ratio (<10% in either ML A or ML B2).

Digital images of H&E-stained whole slides from 12 tumors of TCGA-HNSC were reviewed on 6 September 2022 in the Cancer Digital Slide Archive developed by researchers from the Emory University and Winship Cancer Institute (https://cancer.digitalslidearchive.org/#!/CDSA/hnsc). Selected tumors were classified as ML B by the random forest model and were either PNI− (*n = 6*) according to clinical data or not analyzed (PNI NA).

### 4.7. Analysis of Multiomics Data, Epigenetic Alterations, and Immune Cell Deconvolution

Data for the tumor mutational burden and the fraction of copy number alteration were downloaded from cBioPortal for TCGA-HNSC on 15 September 2020 [65,66]. TCGA-HNSC methylome data [67] were normalized using the R package “watermelon”. Violin plots were created in R using the packages “ggplot2” and “ggpubr”.

For the analysis of the copy number alteration values, a segment mean bigger than 0.5 was defined as a gain and lower than −0.5 as a loss. Copy number alteration summary plots were created by IGV_2.7.2 (Integrative Genomics Viewer) [68]. The COnVaQ online tool was applied for the statistical model using Fisher’s exact test [69].

A somatic mutation analysis was performed using Oncomaps in cBioportal [65,66]. Significant differences in the relative frequency of mutations between ML A and ML B2 were computed by crosstabs and chi-squared tests in R. Results were visualized as bar chart using Excel.

Absolute immune cell scores including different subtypes of B cells (plasma cells, non-plasma B cells), T cells (CD4, CD8), other immune cells (macrophages, NK cells), and stromal cells (endothelium, fibroblasts) of xCell, CIBERSORTx, and Kassandra were downloaded from https://science.bostongene.com/kassandra/downloads (accessed on 21 December 2022). *p*-Values were computed between ML A and ML B2 using a *t*-test.

### 4.8. Gene Set Variation Analysis

Gene set variation analysis (GSVA) scores were computed using the R package “gsva”. The Hallmark gene sets were accessed from the Molecular Signatures Database (MSigDB) [70] on 21 September 2022, and significant differences between ML A and ML B2 were identified with the “limma” package in R.

## 5. Conclusions

In conclusion, we trained a random forest classification model for the risk assessment of perineural invasion that could improve the diagnosis and treatment decision making for primary head and neck squamous cell carcinoma, independent of the quantity of available tumor samples. In contrast to similar approach from previous studies, our model enabled the detection of occult PNI and an extensive validation in different HNSCC cohorts as well as in other solid tumors. With the unprecedented analysis of multiomics data, we were able to provide new insights into the development of perineural invasion at the molecular level. Thus, our machine learning model can also assist future studies in gaining an in-depth knowledge on the basic principles of cellular and molecular alterations driving perineural invasion and to establish new drug targets for innovative therapeutic strategies aiming at the prevention of cancer cell dissemination.

## Data Availability

Publicly available datasets were analyzed in this study. These data can be found here: Table S1.

## Supplementary Materials

The supporting information can be downloaded at: https://www.mdpi.com/article/10.3390/ijms24108938/s1.

## Abbreviations

ANOVA	Analysis of variance
AUC	Area under the curve
CESC	Cervical squamous cell carcinoma
CNA	Copy number alteration
CDKN2A	Cyclin-dependent kinase inhibitor 2A
COAD	Adenocarcinoma of the colon
DEGs	Differentially expressed genes
DNA	Deoxyribonucleic acid
DSS	Disease-specific survival
EMT	Epithelial-to-mesenchymal transition
GSVA	Gene set variation analysis
HNSCC	Head and neck squamous cell carcinoma
HPV	Human papilloma virus
HR	Hazard ratio
IFNK	Interferon kappa
LASCC	Laryngeal squamous cell carcinoma
LUAD	Adenocarcinoma of the lung
ML	Machine learning
MSigDB	Molecular Signatures Database
NSD1	Nuclear receptor-binding SET domain protein 1
OPSCC	Oropharyngeal squamous cell carcinoma
OS	Overall survival
OSCC	Oral squamous cell carcinoma
PAAD	Adenocarcinoma of the pancreas
PDAC	Pancreatic ductal adenocarcinoma
PFI	Progression-free interval
PNI	Perineural invasion
RCT	Radiochemotherapy
RNA-seq	RNA-sequencing
ROC	Receiver-operating characteristic
SCC	Squamous cell carcinoma
scRNA-seq	Single-cell RNA sequencing
TCGA	The Cancer Genome Atlas
TCGA-HNSC	Head and neck squamous cell carcinoma of The Cancer Genome Atlas
UMAP	Uniform manifold approximation and projection
