# Supplementary material for: Establishment of a Machine Learning Model for the Risk Assessment of Perineural Invasion in Head and Neck Squamous Cell Carcinoma
Source: Int J Mol Sci. 2023 May 18;24(10):8938. doi: 10.3390/ijms24108938 (PMC10218829; doi:10.3390/ijms24108938)
Supplement: Supplementary file 1 [file ijms-24-08938-s001.zip › Figure legends of Supplemental Figures.pdf]

Figure legends of Supplemental Figures

Supplemental Figure S1: Overview showing the prognostic relevance of perineural invasion for HNSCC and its different primary sites

(**A**) Kaplan-Meier plots for 5-years overall survival (OS, left), disease-specific survival (DSS, middle) and progression-free intervals (PFI, right) for patients of TCGA-HNSC with an annotated PNI-status. Number of patients at risk at the indicated time points are given below. (**B**) Forest plot for OS, DSS and PFI based on univariate Cox regression models for subgroups of oral (OSCC), laryngeal (LASCC) and oropharyngeal (OPSCC) tumors of TCGA-HNSC. [1] reference group.

Supplemental Figure S2: Flow chart visualizing experimental design of our study

Supplemental Figure S3: Identification of PNI-related DEGs for TCGA-HNSC

Volcano plots illustrates DEGs, which were calculated by limma-voom (**A**) or edgeR (**B**), and candidate genes with the significance level of adj. p val. < 0.05 and log2FC <-1, >1 are indicated in green (up in PNI+) and red (down in PNI+). (**C**) Venn diagram demonstrates 60 overlapping DEGs for the limma-voom and edgeR analysis. (**D**) Heatmap shows an unsupervised hierarchical clustering based on gene expression data for the 60 DEGs and demonstrates a cluster of co-expressed muscle-related genes (n =16).

Supplemental Figure S4: Analysis of the PNI-related 44-gene signature with independent HNSCC cohorts, best performing classification models Heatmaps show an unsupervised hierarchical clustering based on gene expression data of the PNI-related 44-gene signature and confirm a classification into cluster A (red) and sub-clusters B1 (blue) and B2 (green) in independent HNSCC cohorts: GSE117973 (A), GSE65868 (B), and GSE41613 (C). (D-F) ROC curves for calculation of a suitable machine learning classification model (random forest, neural network, logistic regression) illustrated by AUCs.

Supplemental Figure S5: Transmission of our machine learning model and our 44-gene signature to other tumor entities demonstrating the relevance of the model

(**A**) Forest plot for overall survival (OS) based on univariate Cox regression models for indicated TCGA cohorts (CESC, LUAD; PAAD, COAD). [1] indicates the reference group. (**B**) Heatmap shows an unsupervised hierarchical clustering of the 44-gene signature based on gene expression data confirming a classification into cluster A (red) and sub-clusters B1 (blue) and B2 (green) in TCGA-CESC.

Supplemental Figure S6 + S7: Differences In copy number alterations between ML A and ML B2: Zoom-in to illustrate the deletions (blue) and amplifications (red) in the comparison between ML A and ML B2, exemplified here by the regions containing hot spots with the lowest p-values (chromosome 1 (**6A**), 7 (**6B**), 13 (**7A**), 16 (**7B**)).

Supplemental Figure S8: Analysis of immune and stromal cells as part of the comprehensive analysis of multi-omics data to show the potential impact distinct immune cell subsets

Spider plot demonstrating statistical significance by showing calculated p-values between ML A and ML B2 for different immune and stromal cells (B cells, T cells, CD4 T cells, Fibroblasts, Endothelium.
