# Supplementary material for: Establishment of a Machine Learning Model for the Risk Assessment of Perineural Invasion in Head and Neck Squamous Cell Carcinoma
Source: Int J Mol Sci. 2023 May 18;24(10):8938. doi: 10.3390/ijms24108938 (PMC10218829; doi:10.3390/ijms24108938)
Supplement: Supplementary file 1 [file ijms-24-08938-s001.zip › Supplemental_Figures.pptx]

## Slide 1
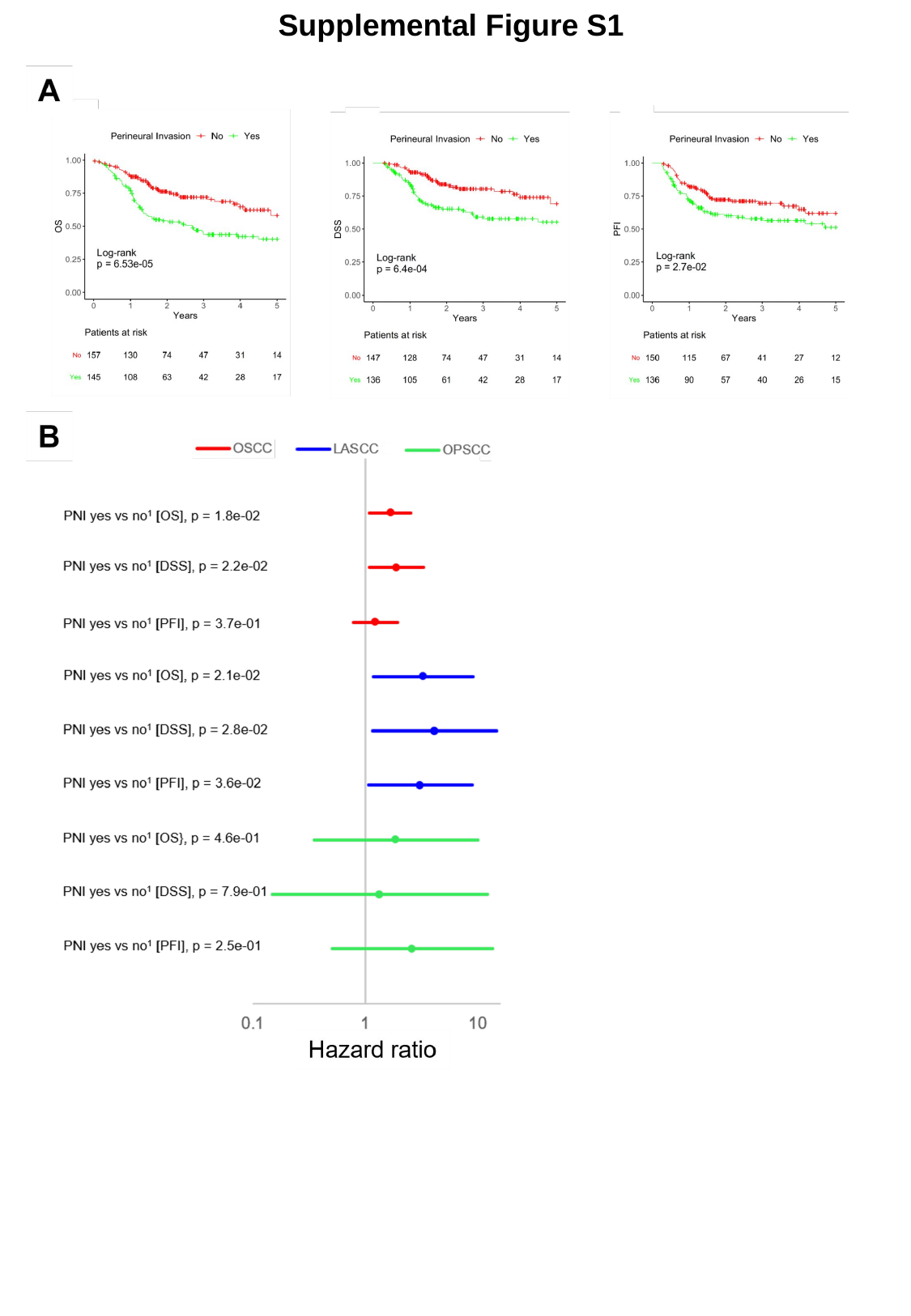

Supplemental Figure S1

## Slide 2
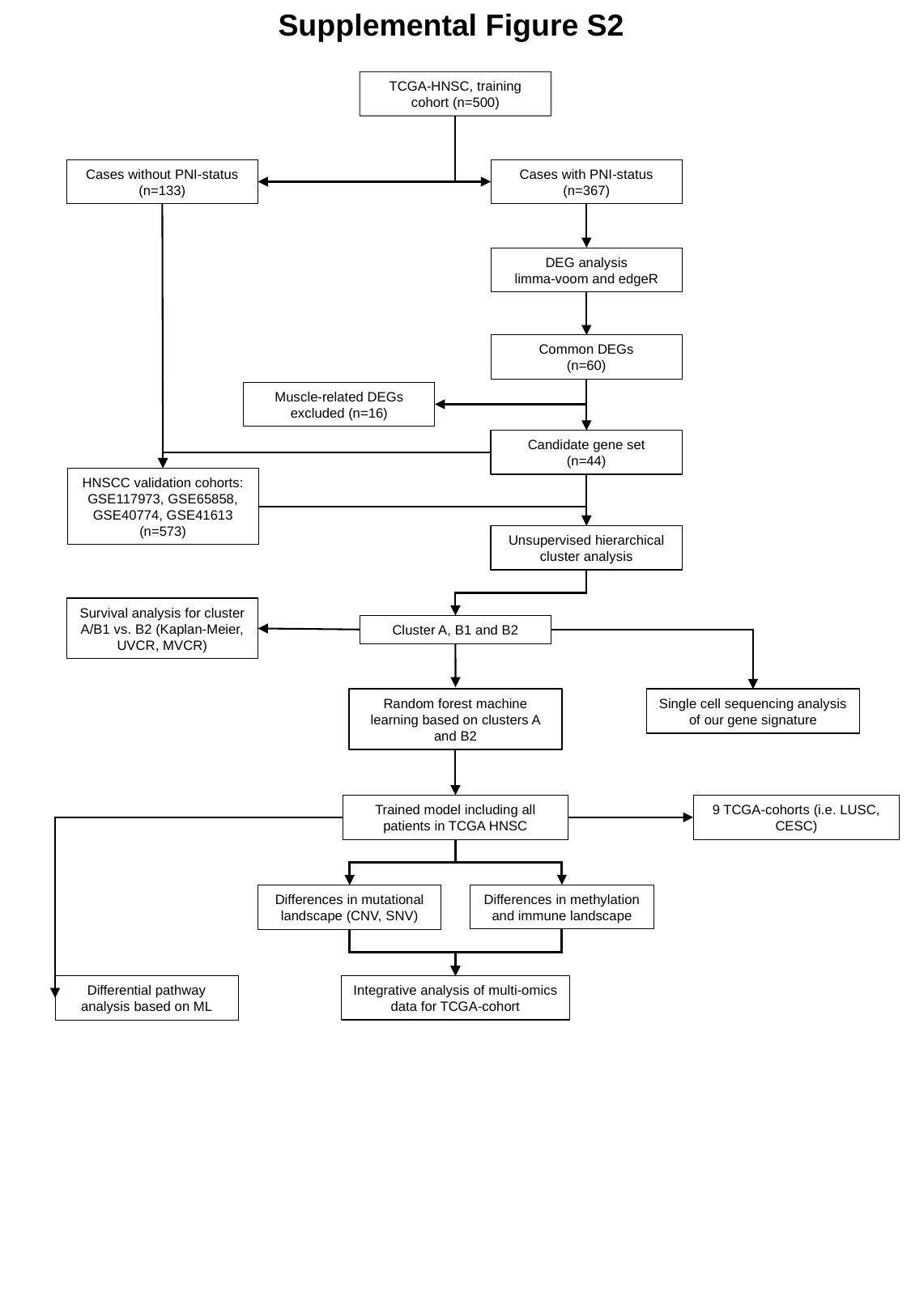

Supplemental Figure S2
TCGA-HNSC, training cohort (n=500)
Cases without PNI-status
(n=133)
Cases with PNI-status
(n=367)
DEG analysis
limma-voom and edgeR
Common DEGs
(n=60)
Muscle-related DEGs excluded (n=16)
Candidate gene set
(n=44)
HNSCC validation cohorts: GSE117973, GSE65858, GSE40774, GSE41613 (n=573)
Unsupervised hierarchical cluster analysis
Survival analysis for cluster A/B1 vs. B2 (Kaplan-Meier, UVCR, MVCR)
Cluster A, B1 and B2
Random forest machine learning based on clusters A and B2
Single cell sequencing analysis of our gene signature
Trained model including all patients in TCGA HNSC
9 TCGA-cohorts (i.e. LUSC, CESC)
Differences in methylation and immune landscape
Differences in mutational landscape (CNV, SNV)
Differential pathway analysis based on ML
Integrative analysis of multi-omics data for TCGA-cohort

## Slide 3
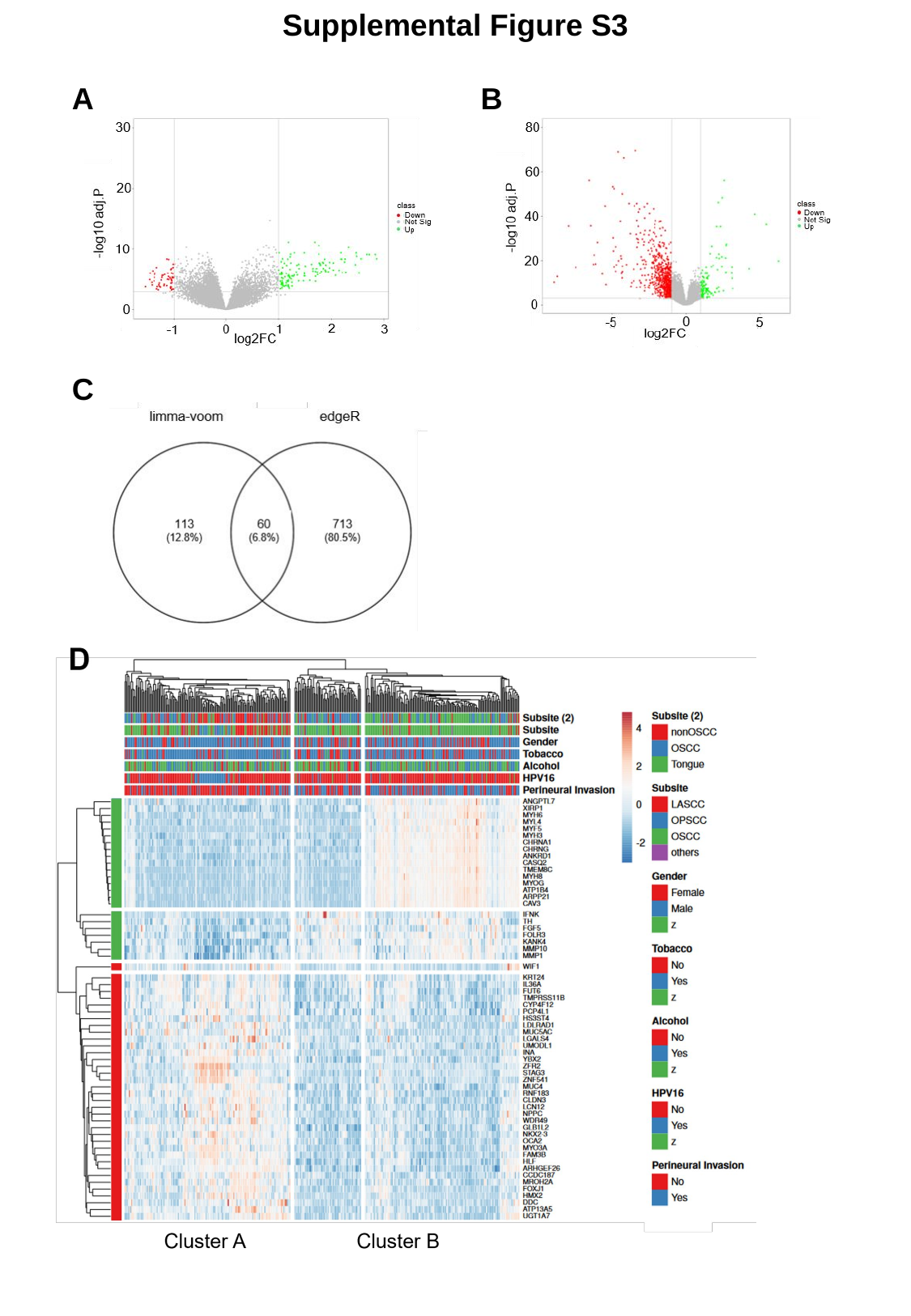

Supplemental Figure S3
A
B
C

## Slide 4
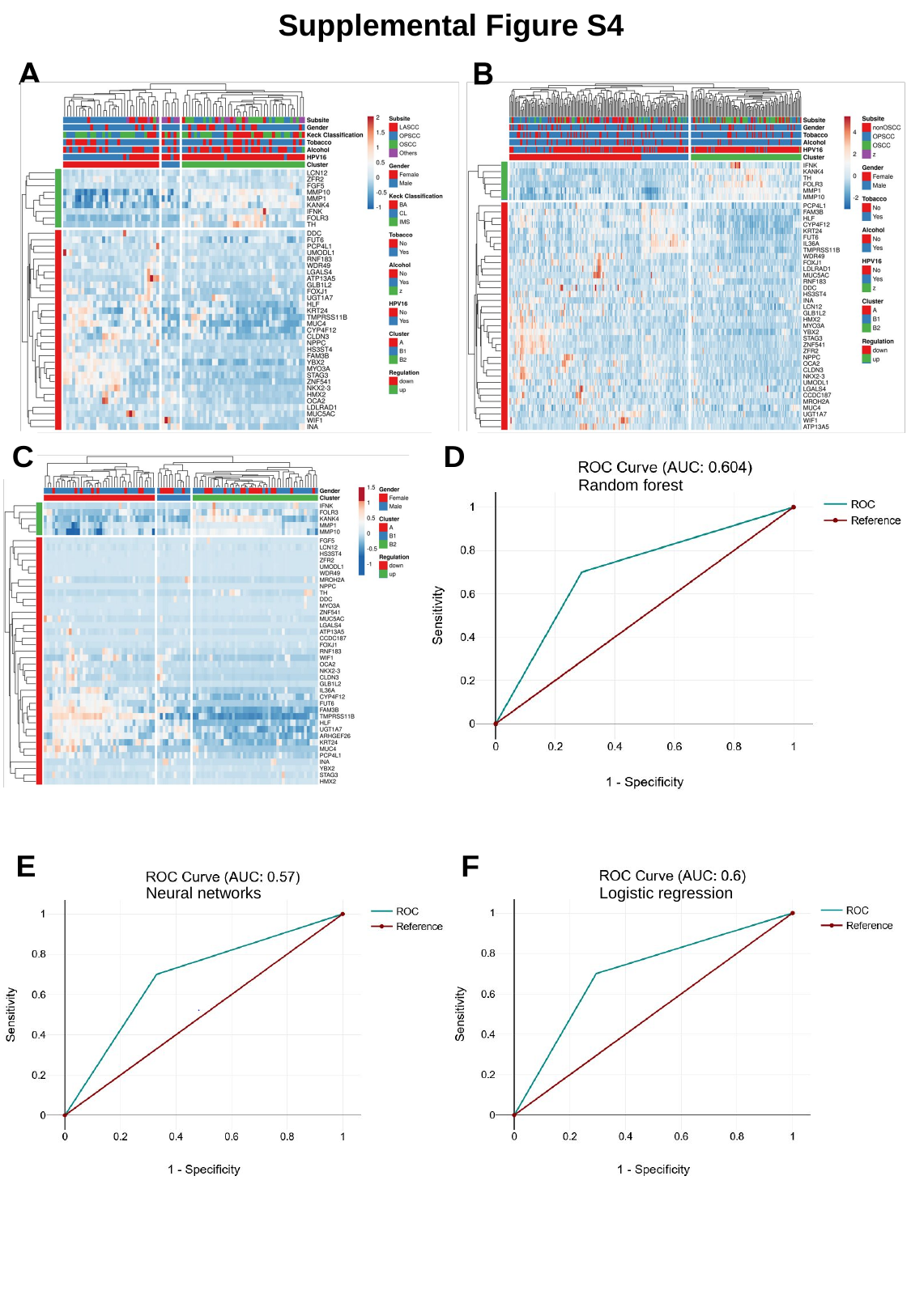

Supplemental Figure S4
C
D
Random forest
F
E
Logistic regression
Neural networks

## Slide 5
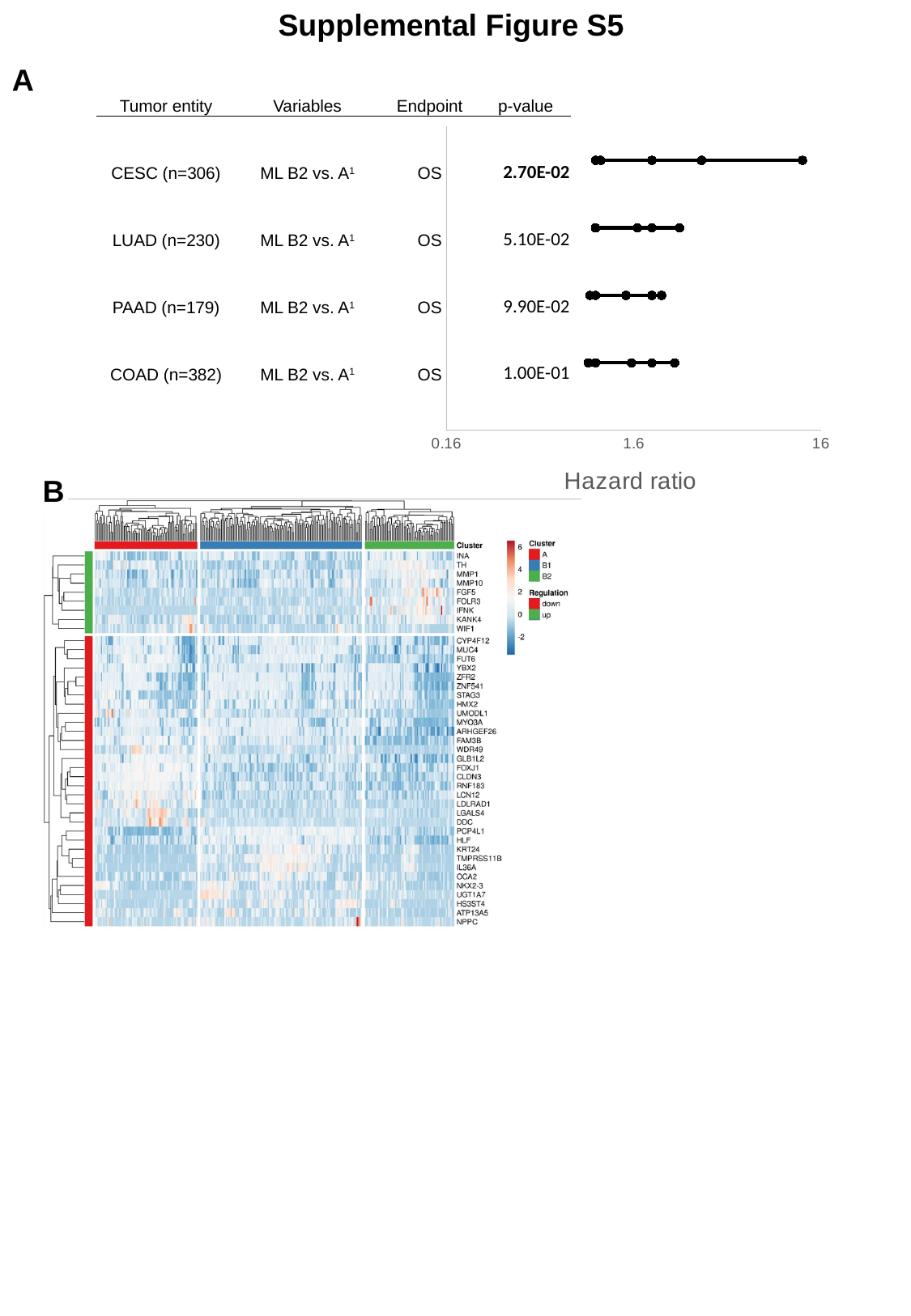

Supplemental Figure S5
| Tumor entity | Variables | Endpoint | p-value |
| --- | --- | --- | --- |
| CESC (n=306) | ML B2 vs. A1 | OS | 2.70E-02 |
| LUAD (n=230) | ML B2 vs. A1 | OS | 5.10E-02 |
| PAAD (n=179) | ML B2 vs. A1 | OS | 9.90E-02 |
| COAD (n=382) | ML B2 vs. A1 | OS | 1.00E-01 |
A
### Chart
| Category | | ESCA | STAD | OV | BLCA | COAD | PAAD | LUAD | CESC | |
|---|---|---|---|---|---|---|---|---|---|---|B

## Slide 6
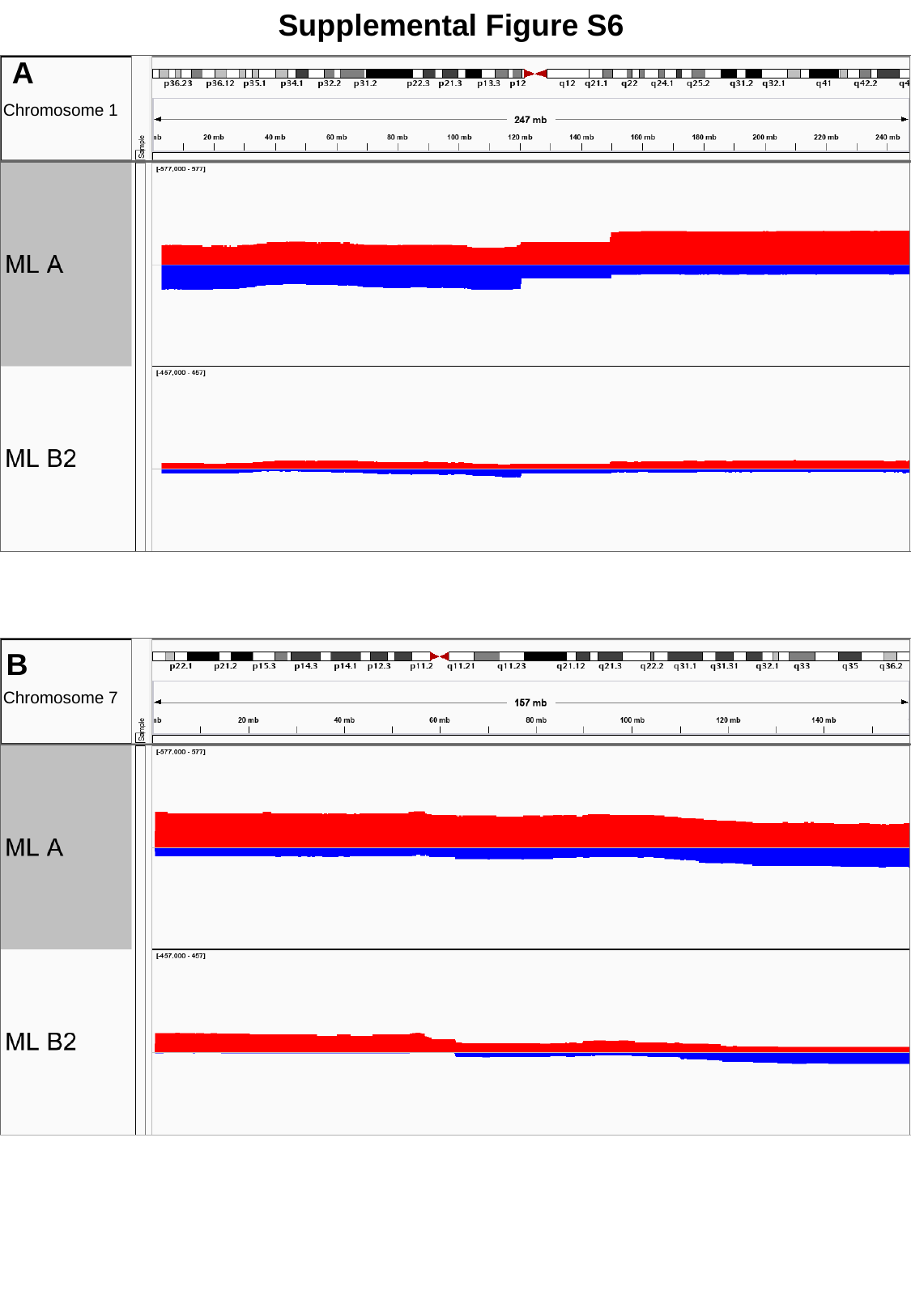

Supplemental Figure S6
A
Chromosome 1
B
Chromosome 7

## Slide 7
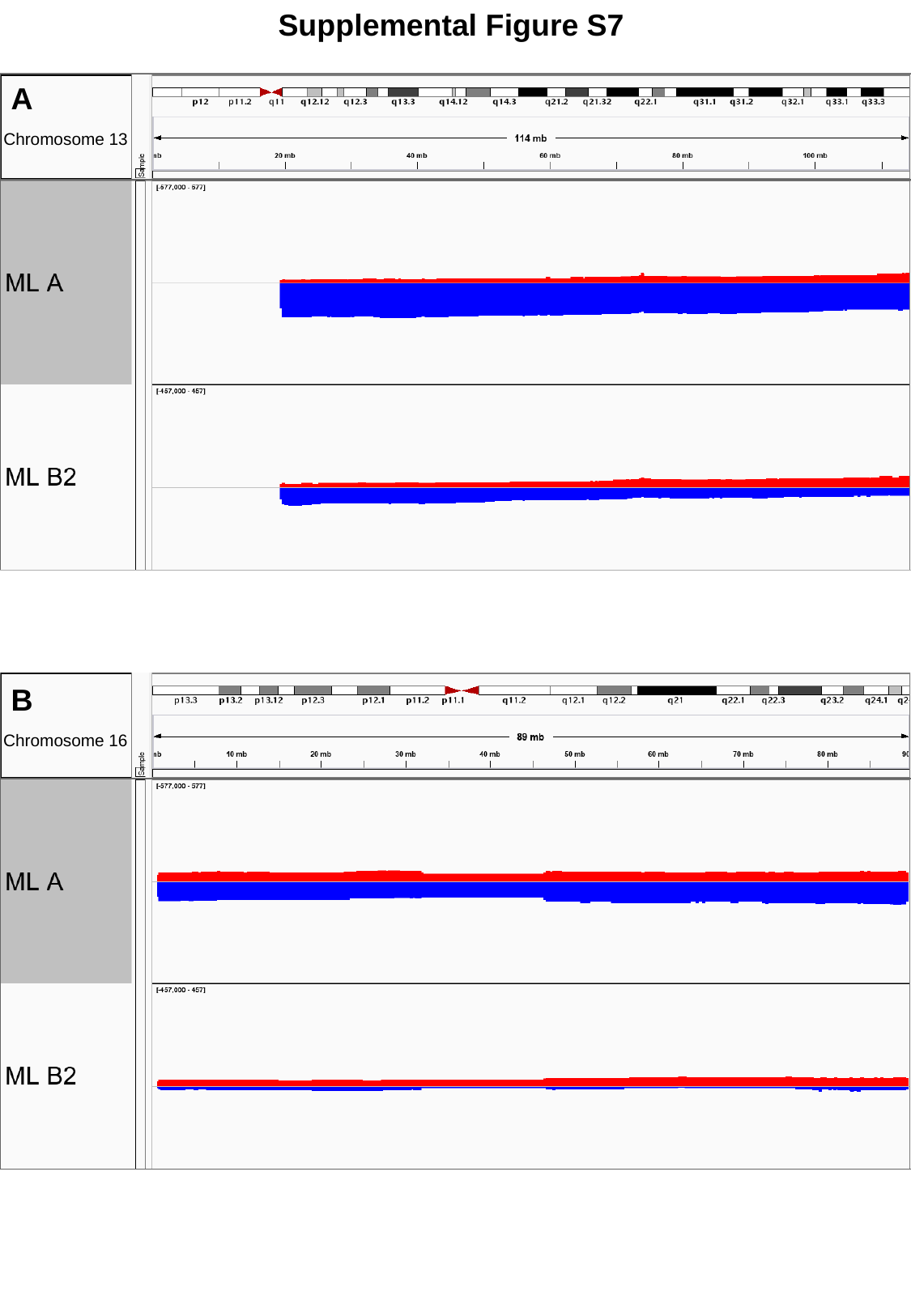

Supplemental Figure S7
A
Chromosome 13
B
Chromosome 16

## Slide 8
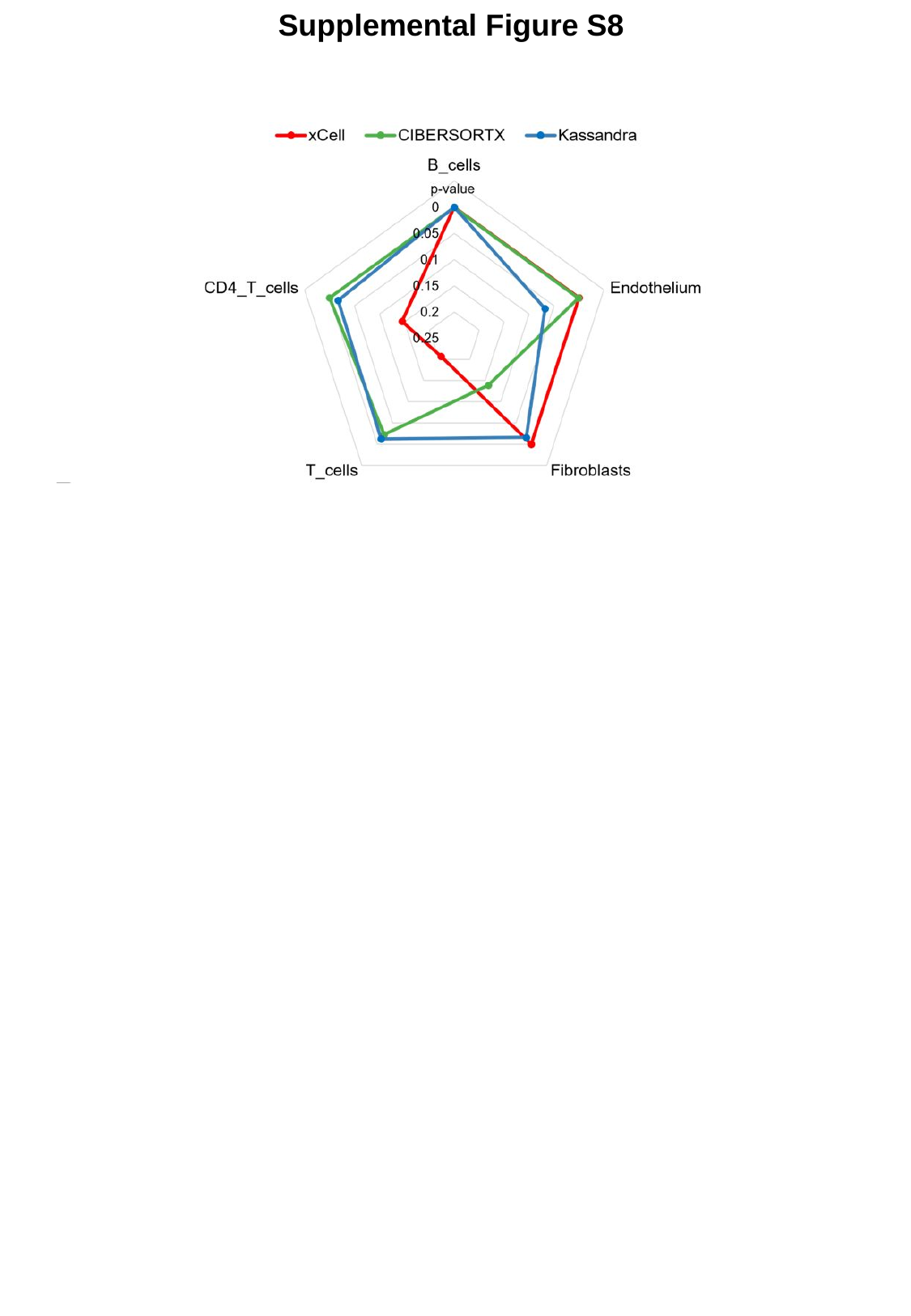

Supplemental Figure S8
